# Expression and characterization of a soluble VEGF receptor 2 protein

**DOI:** 10.1186/2045-3701-4-14

**Published:** 2014-03-11

**Authors:** Wei Liu, Xinyuan Zhang, Ching Song, Shisan Bao, Donna Lai, Jianqiu Mou, Tao Jiang, Ningli Wang

**Affiliations:** 1 Beijing Institute of Ophthalmology, Beijing Tongren Eye Center, Beijing Tongren Hospital, Capital Medical University, Beijing Ophthalmology & Visual Sciences Key Laboratory, Beijing 100730, PR China; 2 University of Science and Technology Beijing, 30 Xueyuan Road, Haidian District, Beijing 100083, P. R. China; 3 Discipline of Pathology, D06, Bosch Institute, the University of Sydney, Sydney, NSW 2006, Australia; 4 Bosch Institute, F13, the University of Sydney, Sydney, NSW 2006, Australia; 5 State Key Laboratory of Molecular Developmental Biology, Institute of Genetics and Developmental Biology (IGDB), No.1 West Beichen Road, Chaoyang District, Beijing 100101, China

**Keywords:** Gene transfection, Truncated soluble protein, Gene construction, Transient transfection

## Abstract

**Objective:**

To clone and express a truncated, soluble vascular endothelial growth factor receptor 2 (sVEGFR2) possessing the combined-functional domains 1–3 and 5 in eukaryotic cells and to test the inhibitory effects of full length VEGFR2 in vivo.

**Results:**

pCMV6-trunctated-rVegfr2 (6100 bp) was successfully cloned. The transfection experiments showed that either pCMV6-truncated-rat-Vegfr2 (pCMV6-truncated-rVegfr2) or pCMV6-rVegfr2 inhibited the expression of intracellular green fluorescent protein, which is usually used as an exogenous transfected reporter gene to determine the transfected efficiency. An analysis of the transfected cells revealed that the amount of full-length VEGFR2 protein in the pCMV6-truncated-rVegfr2 transfected cells was 20% lower than that in the negative control (non-transfected HEK 293 cells). The differences in test results between the transfected and negative control groups were greatest from 24–30 h after transfection; this period was therefore chosen as optimal for collecting culture supernatants. This analysis was highly sensitive for detecting the amount of sVEGFR2 protein expressed and secreted by the cells, and the sVEGFR2 protein content was found to increase by approximately 26% in the transfected cells compared to that in the negative control cells (68.2% ± 1.7% vs. 41.9% ± 2.9%, P = 0.000) and by 18% compared to the negative control cells (68.2% ± 1.7% vs. 50.0% ± 0.5%, P = 0.003). Propidium iodide and Hoechst staining indicated no significant change in the number of HEK293 cells undergoing apoptosis 6 days after pCMV6-trucated-Vegfr2 transfection, compared to the negative control. Soluble VEGFR2 produced by pCMV6-truncated-rVegfr2 inhibited full-length VEGFR2 protein expression in the cell membrane.

**Conclusions:**

This study employed a eukaryotic system to express sVEGFR2. The use of transient transfection technology greatly improved transfect efficiency. Recombinant sVEGFR2 inhibited the effect of endogenous full-length VEGFR2 but was not cytotoxic.

## Background

Vascular endothelial growth factor A (VEGFA), a polypeptide growth factor, originally discovered as a permeability factor is now accepted as an inducer of angiogenesis and vascular leakage both *in vitro* and *in vivo*[1-3]. VEGF acts by binding with its receptors (VEGFR), promoting receptor dimerization and tyrosine auto-phosphorylation. This leads to a kinase cascade in the cell membrane/cytoplasm that transmits a signal to the cell nucleus, inducing a series of changes. VEGFRs are trans-membrane proteins comprised of seven immunoglobulin-like (Ig) extracellular domains, an intracellular tyrosine kinase domain and a trans-membrane sequence.

Members of the VEGFR family include VEGFR1, VEGFR2, and VEGFR3. Among these receptors, VEGFR2 is recognized as the key down regulator for VEGFA’s pathological effects [4]. Vascular leakage, angiogenesis and neuronal degeneration are three features of diabetic retinopathy. VEGFA is a critical driver in these processes, acting as a vascular leakage inducer, angiogenesis mediator and neuron protector in diabetic retinopathy. The VEGF-VEGFR2 pathway was found to play a key role in diabetic retinopathy. Blockage of the VEGF-VEGFR2 pathway may represent a novel therapeutic strategy to reduce vascular leakage and angiogenesis in diabetic retinopathy.

We have previously shown that the function of full length Vegfr2 (long form, 1343 aa) encoded by the rat is similar to humans. A C-terminal truncated version of the protein (991 aa) was found to be encoded by a short form of the *vegfr2*gene [3]. We also demonstrated that the long form*VEGFR2* is the predominant receptor for VEGF-A in the rat retina, while in comparison the truncated form of *VEGFR2* is expressed at very low levels in both normal and diabetic retinas. We further found that the long form of VEGFR2 is the predominant mediator of VEGF-A in the pathogenesis of diabetic retinopathy (DR) and can be significantly inhibited by intravitreal steroid treatment. The short form, which cannot be phosphorylated, does not appear to contribute to the pathogenesis of DR. We proposed that the truncated form of Flk-1 could be used clinically as a dominant negative inhibitor of the effects of VEGF [3].

The extracellular segment of VEGFR2 includes the VEGF ligand binding site. More specifically, the VEGFR2 Ig domain 1 is necessary for VEGF binding, while Ig domains 2–3 ensure a tight bond with VEGF. The receptor employs Ig domain 4 to create the active form of the homologous dimer, whereas Ig domains 5–7 are not closely associated with VEGF binding [5]. Based on our previous findings, we aimed to express a new truncated VEGFR2 by digesting a full-length rat Vegfr2-encoding plasmid to produce a fragment that would encode VEGFR2 Ig domains 1–3 and 5.

## Results

### Sub-cloning of plasmid pCMV6-truncated-rat-Vegfr2

**Figure 1:**
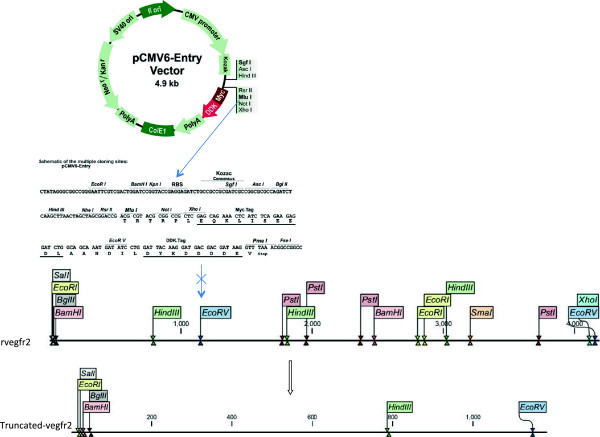
**Schematic of the recombinant plasmid pCMV6-truncated-rVegfr2.** Restriction digestion of the two *EcoRV* restriction sites in the plasmid pCMV6-rVegfr2, which encodes the extracellular reserved VEGFR2 Ig 1–3 and 5 regions. The recombinant plasmid pCMV6-truncated-rVegfr2 was reconstructed after self-ligation of the plasmid vector with T4 DNA ligase. rVegfr2: rat-Vegf-receptor2.

**Figure 2:**
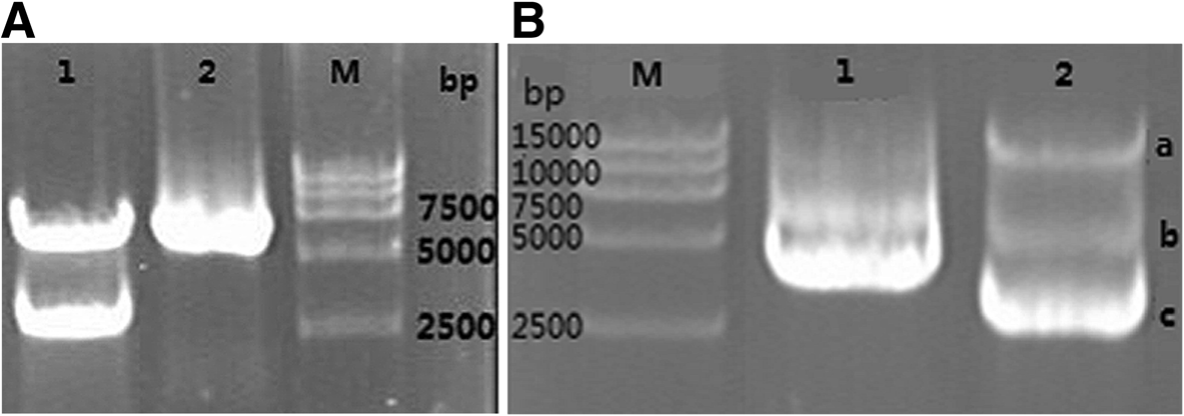
**Electrophoretic analysis of pCMV6-rVegfr2 subcloning.** Lane 1:the two interconnected VEGFR2 enzyme products are 6100 bp and 3006 bp respectively. Lane 2: pCMV-rVegfr2 plasmid. Lane 3: Marker DNA. Lane 1: marker DNA. Lane 2: pCMV-rVegfr2 plasmid. Lane 3: **A**: two interconnected VEGFR2 enzyme products (12200 bp). **B**: an open-loop-shaped pCMV6-truncated-Vegfr2 plasmid. **C**: sub-cloned pCMV6-truncated-rVegfr2 (6100 bp). rVegfr2: rat-Vegf-receptor2.

The pCMV6-rat-Vegfr2 (pCMV6-rVegfr2) plasmid consisted of a 4.2-kb rat-Vegfr2 ORF and a 4.9-kb pCMV6-Entry vector, which were tagged with Myc and DDK, and contained an *EcoRV* restriction enzyme site, respectively. pCMV6-rVegfr2 was truncated with *EcoRV* to remove a 3006-bp gene segment and retain the region that encodes the rVEGFR2 Ig1–3 and5 extracellular soluble proteins, followed by analysis with CLC Sequence Viewer 6.8 software (Figure 1). Following digestion, ligation, transformation and replication in *E.coli*, the plasmids were extracted and screened for the target plasmid via 0.8% agarose gel electrophoresis. Plasmids were truncated with EcoRV to identify the gene fragment of a size that coincided with the expected target gene fragment size. ORF of the *vegfr2* gene and a single 6.1-kb DNA fragment were confirmed by sequencing, indicating that the pCMV6-truncated-rVegfr2 plasmid vector had been constructed successfully (Figure 2).

### Transient transfection

**Figure 3:**
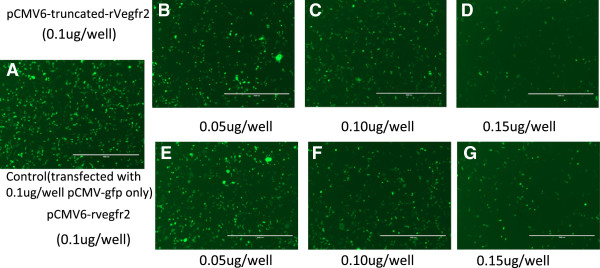
**GFP expression in pCMV6-rVegfr2- or pCMV6-truncated-rVegfr2-transfected HEK293 cells.** 48 hours after HEK293 cells were co-transfected with the pCMV-gfp plasmid and pCMV6-truncated-rVegfr2 or pCMV6-rVegfr2 plasmids, GFP expression was examined under an inverted fluorescence microscope with a 10x objective. The concentration of transfected pCMV-gfp DNA was consistent in all experimental groups (0.10 μg/well and 4 × 10^4^cells/well). **A**, Control group (transfected with 0.1ug/well pCMV-gfp only). **B-D**, transfected pCMV6-truncated-rVegfr2 plasmid with three different concentrations **E-G**, transfected pCMV6-rVegfr2 plasmid with three different concentrations. rVegfr2: rat-Vegf-receptor2. Scale bar: 1000 um.

**Figure 4:**
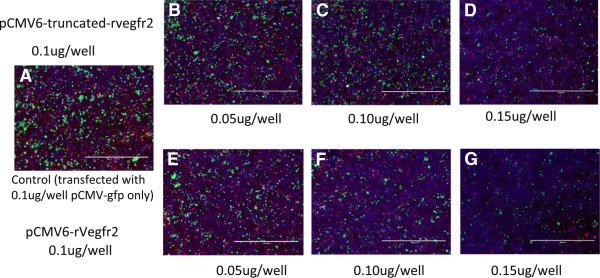
**Transfection efficiencies and apoptotic indices of the pCMV6-rVegfr2- and pCMV6-truncated-rVegfr2- HEK293 cells.** HEK293 cells were co-transfected with pCMV-gfp (0.10 μg/well) and pCMV6-rVegfr 2 or pCMV6-truncated-rVegfr2 plasmids (4 × 10^4^cells/well). PI and Hoechst staining were performed at 72 h after transfection in each experimental group. **A**, Control group (transfected with pCMV-gfp plasmid only). **B-D**, transfected pCMV6-truncated-rVegfr2 plasmid with three different concentrations. **E-G**, the concentrations of transfected pCMV6-rVegfr2 plasmid with three different concentrations. rVegfr2: rat-Vegf-receptor2. Scale bar: 1000 um.

Transient transfection of the exogenous plasmids pCMV6- truncated-rVegfr2 and pCMV6-rVegfr2 into HEK293 cells was performed according to the liposome preparation protocol with a reporter gene pCMV-gfp (0.10 μg/well). To avoid artifact caused by different DNA concentrations, a pCMV/R-luc plasmid was added to the wells to achieve the same concentration. Three groups were generated, the rVegfr2, truncated-rVegfr2 and negative control (non-transfected) groups. The plasmids were transfected into HEK293 cells for 48 h and observed under an inverted fluorescence microscope (Figure 3). To normalize the transfection efficiencies between different groups, GFP protein expression was semi-quantitatively analyzed. For both the rVegfr2 and truncated-Vegfr2 groups, GFP expression decreased with an increase in the desired plasmid concentration. No difference in GFP expression was observed in the rat-Vegfr2 group or truncated-Vegfr2 group at the same time point, however, both in rVegfr2 and truncated-Vegfr2 groups, GFP expression levels decreased over time, it may result from the increased VEGFR2 expression levels over time.propidium iodide (PI) and Hoechst-labeled cells in the different groups were examined 72 h after transfection (Figure 4). Hoechst 33342 brightly stains the condensed chromatin of apoptotic cells and dimly stains the normal chromatin of live cells. So, while Hoechst stained the entire cell population, more intense staining was observed for apoptotic cells and dead cells. PI cannot permeate into live cells, meaning only dead cells were stained red with PI. There were no significant differences in the frequencies of PI-labeled apoptotic cells and the cell densities in the rVegfr2 and truncated-rVegfr2 groups, compared with the negative control group (non-transfected cells), as observed via fluorescent microscopy. The expression of GFP decreased with increasing target plasmid concentrations, suggesting a high transfection efficiency for pCMV6-truncated-rVegfr2.

**Figure 5:**
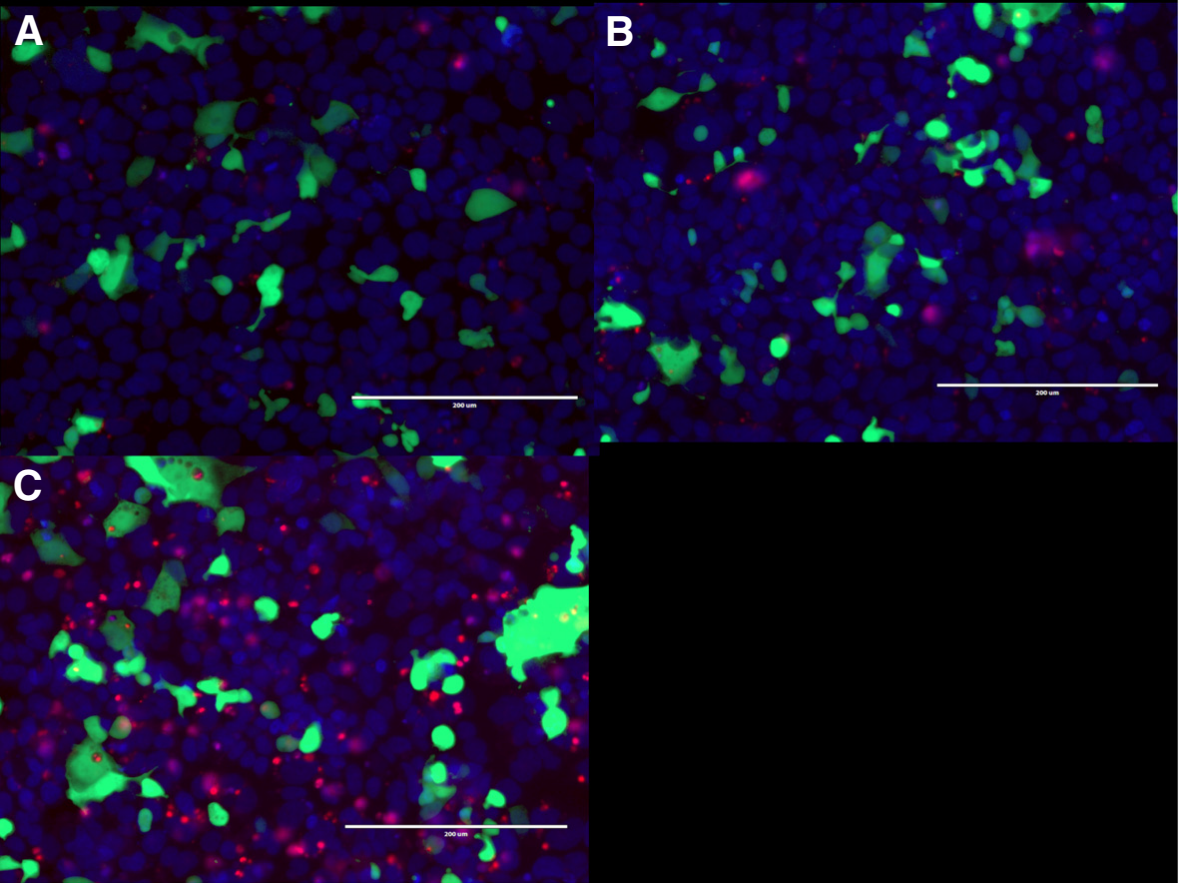
**HEK293 cells were stained and observed at a high magnification (× 20) at 6 days post-transfection.** HEK293 cells were cells cultured and stained with PI and Hoechst dye at 6 days after transfection. The seeding density was consistent in all experimental groups (4 × 10^4^cells/well). **A**, HEK293 cells transfected with pCMV6-rVegfr2 plasmid (0.15 μg/well plus pCMV-gfp plasmid (0.10 μg/well); **B**, HEK293 cells transfected with pCMV6-truncated-rVegfr2 plasmid at 0.15 μg/well plus pCMV-gfp plasmid (0.10 μg/well; and **C**, control group (HEK293 cells transfected with pCMV-gfp plasmid (0.10 μg/well)). rVegfr2: rat-Vegf-receptor2. Scale bar: 200 um.

Large numbers of living cells remained after a 6-day transfection period. GFP expression was also detected in all of the transfected cells (Figure 5). Differences in the transfection rates are summarized in Table 1. There was a significant difference in the transfection rate for the rat-Vegfr2 0.15 group (0.15ug DNA/well), compared with that of the control group (12.3% ± 2.3% vs. 16.8% ± 0.8%, P = 0.022). The difference in transfection rates between the experimental groups (F ratio = 2.192, P = 0.150) was not statistically significant. Transfection efficiencies increased in the HEK293 cells using higher concentrations of pCMV6-truncated-rVegfr2 plasmids. The apoptotic index (AI) is much lower in the 0.05 truncated-rVegfr2, 0.1truncated-rVegfr2 and 0.05 rVegfr2 groups, compared to the control group (22.1% ± 2.2% vs. 29.5% ± 1.0%, P = 0.003; 16.2% ± 2.6% vs. 29.5% ± 1.0%, P = 0.000 and 23.1% ± 3.1% vs. 29.5% ± 1.0%, P = 0.014, respectively). Further, there were no significant differences between the 0.15 truncated-rVegfr2 and 0.10 rVegfr2 group, compared to the control group (AI = 26.4% ± 3.0% vs. 29.5% ± 1.0%, P = 1.000 and 32.2% ± 2.7% vs. 29.5% ± 1.0%, P = 1.000, respectively), indicating that the use of low concentrations of pCMV6-truncated-rVegfr2/pCMV6-rVegfr2 for transfection might attenuate apoptosis. When the plasmid concentration was increased to 0.10, the protective effect disappeared; however, the proteins were not cytotoxic.

**Table 1:** **Difference of transfection rate **$\bar{x}\pm s$

	**Control group (n = 5)**	**Truncated- rVegfr2 2 0.05 group (n = 5)**	**Truncated-rVegfr2 0.l0 group (n = 5)**	**Truncated- rVegfr2 0.l5gourp (n = 5)**	**rVegfr2 0.05group (n = 5)**	**rVegfr2 0.l0group (n = 5)**	**rVegfr2 0.l5group (n = 5)**
**Green fluorescent points**	303.2 ± 19.1	324.8 ± 28.5	387.2 ± 14.5*	216.0 ± 23.3*	272.2 ± 13.1	261.8 ± 17.0*	142.4 ± 15.8*
**Red fluorescent points**	532.2 ± 17.8	452.4 ± 54.6	311.2 ± 20.5	446.6 ± 44.2*	307.8 ± 34.1*	443.0 ± 48.6*	149.2 ± 35.6*
**Blue fluorescent points**	1272.0 ± 49.5	1593.6 ± 116.6*	1640.0 ± 256.9	1252.0 ± 151.3	1029.6 ± 76.9	932.0 ± 55.8*	1030.4 ± 135.9
**Transfection rate of GFP (%)**	16.8 ± 0.8	15.9 ± 1.6	20.1 ± 2.5	12.8 ± 2.3	20.4 ± 1.6	19.1 ± 2.1	12.3 ± 2.3*
**Apoptotic index (%)**	29.5 ± 1.0	22.1 ± 2.2*	16.2 ± 2.6*	26.4 ± 3.0	23.1 ± 3.1*	32.2 ± 2.7	12.8 ± 3.2*

*P < 0.05(Compared with the normal control group) cells were stained with PI and Hoechst.

Green fluorescent points: the number of pCMV-gfp-transfected HEK293 cells.

Transfection efficiency: the number of green fluorescence-positive cells/ the number of living cells (Hoechst-stained cells).

Apoptosis index: the number of PI-positive cells/the number of Hoechst-stained cells.

rVegfr2: Rat-Vegfr2.

### Quantitative analysis of the target protein via ELISA

**Figure 6:**
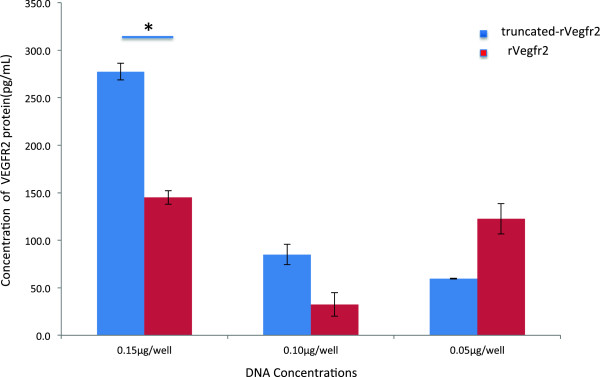
**Effect of the DNA amount on the VEGFR2 concentration in the culture supernatant.** Liposome and plasmid DNA were added at a 1:1 (w/w) ratio, HEK293 were plated at 4 × 10^4^ cells/well, and the DNA concentration ranged from 0–0.15 μg/well.

VEGFR2 protein concentrations in the cell culture supernatants were determined with a VEGFR2-ELISA kit. Supernatant from the serum-free DMEM medium group was used as the negative control. We found that in the truncated-Vegfr2 group, the VEGFR2 protein concentration in the cell culture supernatant decreased along with an increase in the amount of transfected plasmids. In the groups transfected with lower plasmid concentrations (0.05 μg/well and 0.10 μg/well), the amount of sVEGFR2 extracellular protein fragment expression was very low. Therefore, we infer that 0.15 μg/well is the optimal target plasmid transfection concentration. In the rat-Vegfr2 group, there was no association between the concentrations of the transfected pCMV6-rVegfr2 and the extracellular secreted protein (Figure 6).

**Figure 7:**
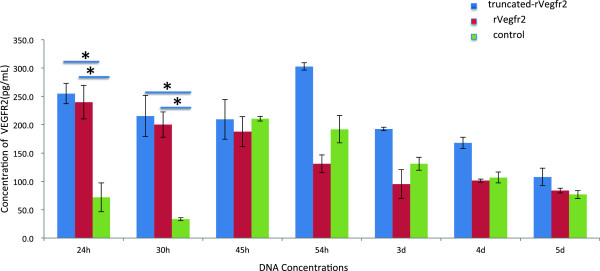
**VEGFR2 expression levels in serum-free DMEM culture supernatants at different time points after transfection.** Liposome and DNA were added in a 1:1 (w/w) ratio to HEK293 (seeding density 4 × 10^4^ cells/well). Truncated-rVegfr2 indicates transfection of 0.15 μg of pCMV6-truncated-rVegfr2 DNA plus 0.10 μg pCMV-gfp/well. rVegfr2 indicates 0.15 μg of transfected DNA plus 0.10 μg pCMV-gfp/well. Control indicates transfection of 0.10 μg pCMV-gfp/well alone. rVegfr2: rat-Vegf-receptor2. *: P < 0.05.

To increase the amount of extracellular-secreted VEGFR2 protein and optimize the incubation time, we collected the cell culture supernatants for ELISA at different time points. Within 24–30 h after transfection, the VEGFR2 protein concentration in the supernatant was higher in the truncated-Vegfr2 and rat-Vegfr2 groups, compared with the negative control group (255.1 ± 36.1 pg/ml vs. 71.9 ± 50.8 pg/ml, P = 0.010 and 215.4 ± 72.2 pg/ml vs. 33.3 ± 5.4 pg/ml, P = 0.010, respectively). The time period from 24–30 h after transfection was selected as the optimal time for cell culture supernatant collection. There were no significant differences between the other time points (P > 0.05), although an increasing trend was observed in the experimental groups, compared with the negative control group (Figure 7). The target protein concentration was higher at 45 h post-transfection than at 24 h and 30 h in the negative control group (210.5 ± 8.5 pg/ml vs. 71.9 ± 50.8 pg/ml and 33.3 ± 5.4 pg/ml, respectively; F ratio = 19.451, P = 0.019). This difference might be caused by interference from the VEGFR2 proteins released from the membrane, and the degradation of the protein over time. At the median time point, the extracellular VEGFR2 protein was somewhat degraded.

**Figure 8:**
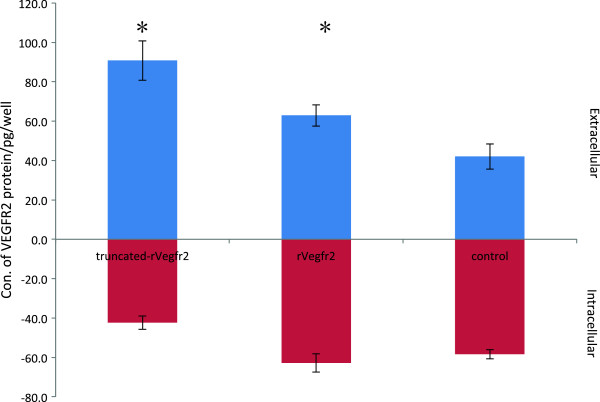
**Intracellular and extracellular VEGFR2 protein concentrations in HEK293 cells under different transfection conditions.** ELISA was used to detect the intracellular and extracellular VEGFR2 protein concentrations in HEK293 cells. Liposomes and DNA were added at a 1:1 (w/w) ratio to HEK293 (seeding density 4 × 10^4^ cells/well). Truncated-Vegfr2 indicates transfection with 0.15 μg of pCMV6-truncated Vegfr2 plasmid plus 0.10 μg pCMV-gfp plasmid/well. Rat-Vegfr2 indicates transfection with 0.15 μg of pCMV6-rVegfr2 plasmid plus 0.10 μg pCMV-gfp/well. Control indicates transfection of 0.10 μg/well pCMV-gfp alone. rVegfr2: rat-Vegf-receptor2. *: P < 0.05.

To determine whether the VEGFR2 protein accumulated inside the transfected cells, we detected intra- and extracellular VEGFR2 protein concentrations via ELISA. The protein concentration in the non-transfected cell group was regarded as the intracellular VEGFR2 protein concentration. The extracellular protein concentration was normalized between the transfected groups and the non-transfected control. The supernatant from serum-free DMEM medium was used as the negative control. The results indicated that the intracellular VEGFR2 concentration was significantly lower in the truncated-rVegfr2 group than in the control group (42.4 ± 3.4 pg/ml vs. 58.3 ± 2.4 pg/ml, P = 0.093). The VEGFR2 concentration was higher in the rat-Vegfr2 group than in the control group (62.9 ± 4.6 pg/ml vs. 58.3 ± 2.4 pg/ml, P = 1.000), but this difference was not significant. There was a significant difference in the intracellular protein concentration between the truncated-rVegfr2 group and the rVegfr2 group (42.4 ± 3.4 pg/ml vs. 62.9 ± 4.6 pg/ml, P = 0.017). The extracellular protein concentrations were higher in the truncated-rVegfr2 and rVegfr2 groups than that in the control group (90.8 ± 10.0 pg/ml vs. 42.0 ± 6.4 pg/ml, P = 0.0001 and 62.9 ± 5.5 pg/ml vs. 42.0 ± 6.4 pg/ml, P = 0.015, respectively). There was a significant difference in the extracellular protein concentration between the truncated-Vegfr2 and rVegfr2 groups (90.8 ± 10.0 pg/ml vs. 62.9 ± 5.5 pg/ml, P = 0.001). The pCMV6-truncated-rVegfr2-transfected cells were stimulated to secrete large amounts of sVEGFR2 protein fragment, resulting in increases in the percentages of extracellular secreted protein production of 18% and 26% over those of the rVegfr2 and negative control groups, respectively (68.2% ± 1.7% vs. 50.0% ± 0.5%, P = 0.003 and 68.2% ± 1.7% vs. 41.9% ± 2.9%, P = 0.000, respectively) (Figure 8).

## Discussion

Several anti-VEGF/VEGFR therapeutic strategies have been developed, including the use of soluble receptors, VEGF and VEGFR-neutralizing antibodies. Other inhibitors that target both VEGF and VEGFRs such as small molecules or antisense oligodeoxynucleotides that inhibit VEGFR signal transduction have also been explored. When used alone or in conjunction with other drugs, these methods can be utilized to treat malignant tumors and other diseases. Research has shown that soluble high-affinity VEGFR2 fragments are therapeutically effective [6,7]. Soluble VEGFR2 fragments can bind endogenous VEGF, thereby blocking the action of VEGF and impeding the binding of VEGFR1, VEGFR2, and VEGFR3 to cell membrane ligands, inhibiting the VEGF-induced signaling pathway, and preventing angiogenesis [8].

VEGFR2, the main functional VEGF receptor, is a type III transmembrane protein kinase that was first discovered in 1991 [9]. The VEGFR2 gene locus is found on chromosome 4q11-q12, and it encodes a receptor with a total length of 1,443 amino acids. VEGFR2 is translated into an intracellular non-glycosylated protein of approximately 150 kDa, that is subsequently repeatedly glycosylated, to yield a mature form of approximately 230 kDa that localizes to the cell membrane.

Our previous work demonstrated that though there are two isoforms of VEGFR2 found in rat, the long form of vegfr2 gene share 99.9% identity of gene sequence with human’s. Given that humans have only a single isoform, we have chosen to express and characterize rat truncated VEGFR2 in this study ahead of further in vivo testing.

Catalysis is facilitated by an extracellular structural domain that contains seven immunoglobulin-like domains, a transmembrane structural domain, and a cytoplasmic tyrosine kinase structural domain. Signal transduction is chiefly mediated by the specific binding of VEGF to the extracellular regions I–III of VEGFR2 [10,11].

Previous studies have only tested a soluble VEGFR2 (sVEGFR2) protein that contained Ig domain 3 (97 amino acids) and have consequently overlooked the regulation of receptor-ligand binding by Ig domains 1 and 2, which reduces the affinity of sVEGFR2 for VEGF. This study employed restriction enzyme digestion in conjunction with the recombinant cloning of a Norway rat Vegfr2 gene restriction fragment into the eukaryotic expression vector pCMV6. The end product was a pCMV6-diagested-Vegfr2 plasmid encoding a form of sVEGFR2 that contained Ig domains 1–3 and 5 of the rat VEGFR2 protein.

Most research on the preparation of sVEGFR proteins has employed recombinant target plasmids to induce target protein expression in an IPTG prokaryotic expression system. However, this process leads to many problems, such as severe cytotoxicity of the expression products, failure to glycosylate the protein product, and failure to achieve correct target protein folding [12,13]. Reports have indicated that most laboratories relied on *Escherichia coli* expression systems to induce sVEGFR1 and sVEGFR2 expression and that the anti-angiogenic efficacies of these expression products was less than 40% [14]. This low level was because *E. coli* usually expresses 50% of the total protein, and rapid bacterial expression leads to either a failure to fold or incorrect folding. These problems are particularly common in proteins that require long expression times such as heterogeneous proteins or those with molecular companions. The transient transfection method ensures that the exogenous genes exist only in free plasmid vectors after target cell transfection. The plasmids do not integrate into the chromosomes. Consequently, the target gene expression products can be obtained in a short time; however, as the cells proliferate, the exogenous genes ultimately disappear. The expression process can continue for several days or up to 2 weeks. This method can greatly shorten the process of recombinant protein production and thus provides a rapid and convenient gene expression method. In addition, we co-transfected, the reporter pCMV-gfp plasmid with the target plasmids for transfection condition optimization.

We initially chose to transfect HEK293 cells in a 48-well plate at 80–90% confluency. However, given the relatively long experimental period, the cells invariably became fully confluent within 24 h of transfection. The cells also underwent apoptosis due to overgrowth during the observation stage, thus influencing subsequent observations and testing. We therefore reduced the required cell density to 50–60% confluency at the time of transfection, although this procedure resulted in lower cell transfection efficiencies and reduced target protein expression. Regarding ELISA analysis, we initially chose to use a VEGFR2 ELISA Kit to test the culture supernatants after the cells had been cultured in DMEM with 10% fetal bovine serum (FBS). However, as the background values were higher in the control group than the experimental group, we concluded that miscellaneous proteins in the FBS had severely interfered with the test results, and therefore, the cultures were performed in serum-free DMEM. Given the need for growth factors in the medium, 1 × 10^−4^ v/v EGF was added to ensure cell survival.

Regarding the pCMV6-sVegfr2 plasmid obtained by sub-cloning, we discovered that differences in transfection efficiencies of pCMV6-truncated-rVegfr2 or pCMV6-rVegfr2 in the cells caused different green fluorescent protein expression levels in the cells. This discrepancy might have been due to inhibited GFP expression caused by exogenous target plasmid (pCMV6-truncated-rVegfr2 or pCMV6-rVegfr2) transfection and expression. While the exogenous plasmid transfection efficiencies were largely identical among the experimental groups, the transfection of HEK293 cells with large amounts of pCMV6-rVegfr2 led to reduced transfection efficiency. Transfection with a lower concentration of either pCMV6-truncated-rVegfr2 or pCMV6-rVegfr2 had a protective effect on the cells, inhibiting apoptosis. When the target plasmid concentration was increased, this protective effect was lost but the expressed protein was not cytotoxic. While transfection with pCMV6-truncated-Vegfr2 induced HEK293 cells to express truncated-VEGFR2 protein, the amount of pCMV6-rVegfr2 transfected into the cells did not closely correlate with the amount of truncated-VEGFR-2 secreted into the culture medium. The differences in the results between the transfection and control groups were greatest from 24–30 h after transfection. This period was therefore chosen as the optimal time for culture supernatant collection. Cells in the control and study groups exhibited poor growth at 45 h after plasmid transfection. This may have been due to the release of membrane-associated VEGFR2 protein into the culture medium; the protein fragments were thus degraded as the culture time increased, and the secreted sVEGFR2 in the culture medium tended to decompose. Transient transfection with different exogenous plasmids influenced sVEGFR2 protein expression and secretion both inside and outside cells. pCMV6-truncated-rVegfr2-transfected cells expressed and secreted both the soluble VEGFR2 protein and soluble VEGFR2 protein fragments at high efficiencies. The amount of sVEGFR2 protein secreted by cells in the sVEGFR2 group, as a percentage of all intracellular and extracellular VEGFR2 protein, increased by nearly 10%, compared to that secreted by cells in the soluble VEGFR2 group, and by more than 20% compared to that secreted by cells in the control group. We propose that pCMV6-rVegfr2 induced copious VEGFR2 protein expression on the cell membrane; however, the limited ability of the cell membrane to bind this protein allowed the excess release of receptor protein fragments into the culture medium. In contrast, VEGFR2 protein expression on the cell membrane was lower in the control group, and the positive results obtained when testing the culture supernatant might have been caused by the release of VEGFR2 receptor protein fragments from the cell membrane when the cells underwent apoptosis.

Given the limitations on the number of cells per well, the reduced plate coverage after transfection, and the degradation of sVEGFR2 during the culture process, this study obtained a relatively low target protein yield. Subsequent studies should aim to inhibit protein degradation, ease the cell culture and protein expression restrictions, and perform specific testing of the sVEGFR2 protein with the ultimate goal of purifying and testing its biological activity.

After transfecting cells with pCMV2-trucated-rVegfr2, we were able to obtain serum-free DMEM culture supernatants containing VEGFR2 protein fragments that bound to VEGF. This work will provide a basis for the development of soluble receptors that target endogenous VEGF and treatments for diabetic retinopathy that target VEGFR2.

## Materials and methods

### Cell lines and reagents

HEK293 cells were purchased from the Cancer Institute of the Chinese Academy of Medical Sciences Cell Bank. The *E. coli* DH5α strain was from our laboratory. The restriction endonuclease *EcoRV* and T4 DNA Ligase were obtained from Takara China (TAKARA Biotechnology Co., LTD., Dalian, Shangdong China). pCMV6-rVegfr2 was constructed by ORIGENE (ORIGENE, China). The mini-plasmid extraction kit, plastic recycling plasmid extraction kit, and endotoxin-free plasmid extraction kit were purchased from TIANGEN China (Tiangen, Beijing, China). Dulbecco’s modified Eagle’s medium (DMEM) powder and fetal bovine serum (FBS) were purchased from Gibco Life Technologies (New York, NY, USA). Epidermal growth factor (EGF) was obtained from Sino Biological, Inc (Beijing, China). The rat VEGFR2 ELISA Kit was purchased from Shanghai Yuanye Biological, Inc (Shanghai, China). Deionized distilled water was utilized throughout the entire study.

## Methods

### Construction of the recombinant plasmid pCMV6-truncated-rVegfr2

The plasmid pCMV6-rVegfr2 was constructed according to the nucleotide sequence published in GenBank (GenBank accession number NM_013062.1). pCMV6-rVegfr2 was truncated with the restriction endonuclease *EcoRV* in a mixture containing 2.5 μl of EcoRV, 5 μl of 10× H Buffer, 30 μl of pCMV6-rVegfr2 DNA, and 12.5 μl of nuclease-free water; the digestion mixture was incubated overnight at 37ºC. The product was detected using 0.8% agarose gel electrophoresis. The 6100 bp fragment in the gel was recycled and extracted. pCMV6-truncated-rVegfr2 (which encodes VEGFR2 Ig domains 1–3 and 5) was recombined using T4 DNA Ligase in a mixture containing 2 μl of T4 DNA Ligase, 40 μl of recovered plasmid DNA, 5 μl of 10× buffer, and 3 μl of deionized distilled water, the mixture was incubated at 4ºC overnight. The recombinant vector was then transformed into the cloning host strain *E. coli* DH5α. Ligation mixture was then transformed into *E. coli*. The recombinant plasmids were purified and detected using 0.8% agarose gel electrophoresis. A large amount of the recombinant vector pCMV6-truncated-rVegfr2 was extracted using an endotoxin-free plasmid extraction kit according to the manufacturer’s instructions, and the OD260/OD280 ratio was measured with a UV spectrophotometer.

### Transient transfection and expression

HEK293 cells were cultured in DMEM (17.8 mM NaHCO3, 10% FBS, 1/10^4^ v/v of EGF) at 37ºC in 5% CO_2_. The cells were plated at a density of 4 × 10^4^ cells/well in 48-well plates and incubated for 24 h. The plasmids pCMV6-truncated-rVegfr2/pCMV6-rVegfr2, pCMV-gfp, and pCMV/R-luc (expressing ocean intestinal lumen luciferase) were transiently transfected into HEK293 cells and the culture medium was discarded and replaced with 250 μl/well of serum-free DMEM medium containing 1/10^4^ v/v EGF, the cells were incubated at 37°C for 48 h to 6 d. The cells were stained with PI and Hoechst dye and observed under an inverted fluorescence microscope.

### ELISA

HEK293 cells were transfected with different plasmids and incubated at 37ºC 5% CO_2_ for 72 h, as described previously [15]. The supernatants were carefully collected and centrifuged at 3,000 rpm for 10–20 min to allow detection of the extracellular VEGFR2 protein concentrations with Rat VEGFR2 protein ELISA kits. ELISA absorbance was measured at 450 nm.

### Quantification and statistical analysis

Fluorescent cells were counted at 40× magnification and analyzed with Image J software. The quantitative analyses were performed at least three times. All data are presented as means ± SEM. A one-way ANOVA was performed for the statistical analysis (SPSS, version 16.0; IBM, USA). The mean values for the groups were compared with ANOVA, and pairwise comparisons between the groups were performed with the LSD-t test. A P-value < 0.05 was considered significant.

## Abbreviations

CMV: Cytomegalovirus; DMEM: Dulbecco’s modified eagle medium; DR: Diabetic retinopathy; E. coli: Escherichia coli; EGF: Epidermal growth factor; ELISA: Enzyme linked immunosorbent assay; FBS: Fetal bovine serum; GFP: Green fluorescent protein; HEK293: Human embryonic kidney 293 cells; Hoechst 33342: Hoechst 33342 solution; Ig: Immunoglobulin; OD: Optical density; ORF: Open reading frame; PI: Propidium Iodide; rVegfr2: rat Vegf receptor 2; sVEGFR2: soluble vascular endothelial growth factor receptor 2; VEGF: Vascular endothelial growth factor; VEGFR: Vascular endothelial growth factor receptor.

## Competing interests

The authors declare that they have no competing interests.

## Authors’ contribution

WL, XZ, CHS and TJ, SB, DL, JM, and NW have been involved in drafting the manuscript. All authors read and approved the final manuscript.

## References

[B1] Senger DR Connolly DT Van de Water L Feder J Dvorak HF Purification and NH2-terminal amino acid sequence of guinea pig tumor-secreted vascular permeability factor Cancer Res 1990 50 1774 1778 2155059

[B2] Cooper ME Vranes D Youssef S Stacker SA Cox AJ Rizkalla B Casley DJ Bach LA Kelly DJ Gilbert RE Increased renal expression of vascular endothelial growth factor (VEGF) and its receptor VEGFR-2 in experimental diabetes Diabetes 1999 48 2229 2239 10535459 10.2337/diabetes.48.11.2229

[B3] Zhang X Chen M Gillies MC Two isoforms of Flk-1 transcripts in early diabetic rat retinas Curr Eye Res 2012 37 73 79 22121831 10.3109/02713683.2011.629766

[B4] Ferrara N Gerber HP LeCouter J The biology of VEGF and its receptors Nat Med 2003 9 669 676 12778165 10.1038/nm0603-669

[B5] Stuttfeld E Ballmer-Hofer K Structure and function of VEGF receptors IUBMB Life 2009 61 915 922 19658168 10.1002/iub.234

[B6] Jacobi J Tam BY Wu G Hoffman J Cooke JP Kuo CJ Adenoviral gene transfer with soluble vascular endothelial growth factor receptors impairs angiogenesis and perfusion in a murine model of hindlimb ischemia Circulation 2004 110 2424 2429 15477417 10.1161/01.CIR.0000145142.85645.EA

[B7] Kou B Li Y Zhang L Zhu G Wang X Li Y Xia J Shi Y In vivo inhibition of tumor angiogenesis by a soluble VEGFR-2 fragment Exp Mol Pathol 2004 76 129 137 15010291 10.1016/j.yexmp.2003.10.010

[B8] Zhang X Bao S Hambly BD Gillies MC Vascular endothelial growth factor-A: a multifunctional molecular player in diabetic retinopathy Int J Biochem Cell Biol 2009 41 2368 2371 19646547 10.1016/j.biocel.2009.07.011

[B9] Matthews W Jordan CT Gavin M Jenkins NA Copeland NG Lemischka IR A receptor tyrosine kinase cDNA isolated from a population of enriched primitive hematopoietic cells and exhibiting close genetic linkage to c-kit Proc Natl Acad Sci USA 1991 88 9026 9030 1717995 10.1073/pnas.88.20.9026 PMC52644

[B10] Roskoski R Jr VEGF receptor protein-tyrosine kinases: structure and regulation Biochem Biophys Res Commun 2008 375 287 291 18680722 10.1016/j.bbrc.2008.07.121

[B11] Li H Cao W Chen Z Acheampong DO Jin H Li D Zhang J Wang M The antiangiogenic activity of a soluble fragment of the VEGFR extracellular domain Biomed Pharmacother 2013 67 599 606 23906761 10.1016/j.biopha.2013.06.001

[B12] Leopold AV Baklaushev VP Pavlov KA Chekhonin VP Expression of a recombinant extracellular fragment of human vascular endothelial growth factor receptor VEGFR1 in E. coli Bull Exp Biol Med 2011 151 347 352 22451884 10.1007/s10517-011-1327-7

[B13] Neufeld G Cohen T Gengrinovitch S Poltorak Z Vascular endothelial growth factor (VEGF) and its receptors The FASEB Journal 1999 13 9 22 9872925

[B14] Farnia P Bandehpour M Ghanavi J Kazemi B Cloning and expression of soluble vascular endothelial growth factors receptor-1 (sFlt-1) fragments in CHO-K1 Int J Clin Exp Med 2013 6 773 778 24179570 PMC3798212

[B15] Baldi L Muller N Picasso S Jacquet R Girard P Thanh HP Derow E Wurm FM Transient gene expression in suspension HEK-293 cells: application to large-scale protein production Biotechnol Prog 2005 21 148 153 15903252 10.1021/bp049830x

